# Global Comparative Genomics of *Stenotrophomonas maltophilia* Reveals Cryptic Species Diversity, Resistome Variation, and Population Structure

**DOI:** 10.3390/life16010158

**Published:** 2026-01-17

**Authors:** Ei Phway Thant, Chollachai Klaysubun, Sirikan Suwannasin, Thitaporn Dechathai, Kamonnut Singkhamanan, Thunchanok Yaikhan, Nattarika Chaichana, Rattanaruji Pomwised, Monwadee Wonglapsuwan, Sarunyou Chusri, Komwit Surachat

**Affiliations:** 1 Department of Biomedical Sciences and Biomedical Engineering, Faculty of Medicine, Prince of Songkla University, Songkhla 90110, Thailand; 6510330008@email.psu.ac.th (E.P.T.);; 2 Division of Biological Science, Faculty of Science, Prince of Songkla University, Songkhla 90110, Thailand; 3 Division of Infectious Diseases, Department of Internal Medicine, Faculty of Medicine, Prince of Songkla University, Songkhla 90110, Thailand

**Keywords:** *Stenotrophomonas maltophilia* complex, resistome, mobilome

## Abstract

**Background:** *Stenotrophomonas maltophilia* is an increasingly important multidrug-resistant opportunistic pathogen frequently isolated from clinical, environmental, and plant-associated niches. Despite its medical relevance, the global population structure, species-complex boundaries, and genomic determinants of antimicrobial resistance (AMR) and ecological adaptation remain poorly resolved, partly due to inconsistent annotations and fragmented genomic datasets. **Methods:** Approximately 2400 genome assemblies annotated as *Stenotrophomonas maltophilia* were available in the NCBI Assembly database at the time of query. After pre-download filtering to exclude metagenome-assembled genomes and atypical lineages, 1750 isolate genomes were retrieved and subjected to stringent quality control (completeness ≥ 90%, contamination ≤ 5%, ≤500 contigs, N50 ≥ 10 kb, and ≤1% ambiguous bases), yielding a final curated dataset of 1518 high-quality genomes used for downstream analyses. Genomes were assessed using CheckM, annotated with Prokka, and compared using average nucleotide identity (ANI), pan-genome analysis, core-genome phylogenomics, and functional annotation. AMR genes, mobile genetic elements (MGEs), and metadata (source, host, and geographic origin) were integrated to assess lineage-specific genomic features and ecological distributions. **Results:** ANI-based clustering resolved the *S. maltophilia* complex into multiple distinct genomospecies and revealed extensive misidentification of publicly deposited genomes. The pan-genome was highly open, reflecting strong genomic plasticity driven by accessory gene acquisition. Core-genome phylogeny resolved well-supported clades associated with clinical, environmental, and plant-related niches. Resistome profiling showed widespread intrinsic MDR determinants, with certain lineages enriched for efflux pumps, β-lactamases, and trimethoprim–sulfamethoxazole resistance markers. MGE analysis identified lineage-specific integrative conjugative elements, prophages, and transposases that correlated with source and geographic distribution. **Conclusions:** This large-scale analysis provides the most comprehensive genomic overview of the *S. maltophilia* complex to date. Our findings clarify species boundaries, highlight substantial taxonomic misannotation in public databases, and reveal lineage-specific AMR and mobilome patterns linked to ecological and clinical origins. The curated dataset and evolutionary insights generated here establish a foundation for global genomic surveillance, epidemiological tracking, and future studies on the evolution of antimicrobial resistance in *S. maltophilia*.

## 1. Introduction

*Stenotrophomonas maltophilia* is a globally distributed, Gram-negative, non-fermenting bacterium increasingly recognized as an opportunistic pathogen of considerable clinical importance [1,2]. Although historically regarded as an environmental organism inhabiting soil, water systems, and plant rhizospheres [3], *S. maltophilia* has emerged as a significant cause of hospital-acquired infections, particularly in immunocompromised individuals and patients with prolonged hospital stays [3]. Clinical manifestations range from respiratory tract infections and bacteremia to device-associated infections, and therapeutic management is challenging due to the organism’s intrinsic and acquired multidrug resistance (MDR) profiles [4]. The World Health Organization recently designated *S. maltophilia* as a high-priority MDR pathogen, underscoring the urgent need to better understand its evolution, population structure, and genomic mechanisms of pathogenicity and antimicrobial resistance [5].

A major barrier to understanding *S. maltophilia* biology is its extensive genomic diversity and complex taxonomy. Increasing evidence indicates that *S. maltophilia* is not a single homogeneous species but part of a broader *S. maltophilia* species complex comprising multiple cryptic genomospecies distinguished by average nucleotide identity (ANI), phylogenomic, and functional traits [6,7,8]. Public genome databases contain numerous misannotated assemblies, and conventional identification methods used in clinical microbiology laboratories often fail to discriminate between closely related species within the genus [9]. This taxonomic ambiguity has hindered accurate epidemiological surveillance, obscured lineage-specific resistance patterns, and complicated the interpretation of comparative genomic studies.

Several previous genomic investigations have examined aspects of *S. maltophilia* evolution, including small-scale pan-genome analyses, resistance gene profiling, and comparative studies of clinical and environmental isolates [10,11,12,13,14]. However, these studies typically relied on limited genome sets, used inconsistent quality-filtering criteria, or lacked comprehensive integration of ecological, geographic, and genomic data. As a result, fundamental questions remain unresolved: How many distinct genomospecies constitute the *S. maltophilia* complex? What is the global population structure across clinical, environmental, and plant-associated niches? How do antimicrobial resistance genes and mobile genetic elements vary among lineages? And to what extent do source and geography shape genomic diversity?

Despite increasing availability of *Stenotrophomonas* genome sequences, several fundamental challenges remain conceptually distinct and require integrated resolution. First, substantial taxonomic ambiguity persists within the *S. maltophilia* complex due to historical misannotation, inconsistent species definitions, and the presence of cryptic lineages, complicating comparative analyses and epidemiological interpretation. Second, the ecological and geographic distribution of genetically distinct lineages across clinical, environmental, and plant-associated reservoirs remains incompletely characterized at a global scale. Third, the evolutionary dynamics linking antimicrobial resistance, mobile genetic elements, and lineage structure remain poorly resolved, particularly in the context of heterogeneous sampling and fragmented genome assemblies.

Beyond expanding dataset scale, the present study addresses these gaps through a unified analytical framework integrating consistent genome re-annotation, ANI-based species delineation, large-scale pan-genome reconstruction, joint mobilome–resistome profiling, rigorous metadata standardization, and public release of a fully reproducible curated genome resource.

The rapid expansion of publicly available whole-genome sequences provides an unprecedented opportunity to address these knowledge gaps [14]. At the time of database interrogation, approximately 2400 genome assemblies annotated as *Stenotrophomonas maltophilia* were available in the NCBI repository. After applying pre-download filtering to exclude metagenome-assembled genomes and atypical lineages, 1750 genomes were retrieved for analysis and subsequently subjected to quality control, yielding a final dataset of 1518 genomes. The curated genomes were analyzed using a comprehensive comparative framework, including ANI-based species delineation, pan-genome reconstruction, core-genome phylogeny, resistome and mobilome profiling, and ecological association testing.

Through this integrated framework, we aimed to (i) resolve species boundaries within the *S. maltophilia* complex, (ii) characterize global genomic diversity and population structure, (iii) define the distribution of antimicrobial resistance genes (ARGs) and mobile genetic elements (MGEs) across ecological and geographic contexts, and (iv) establish a reference-quality curated genomic resource to support future epidemiological and evolutionary research.

By integrating large-scale comparative genomics with high-resolution functional and ecological analyses, this study provides the most extensive genomic characterization of the *S. maltophilia* complex to date and advances current understanding of its taxonomy, evolution, and antimicrobial resistance dynamics.

## 2. Materials and Methods

### 2.1. Genome Retrieval and Dataset Construction

All publicly available genomes annotated as *Stenotrophomonas maltophilia* were retrieved from the NCBI Assembly database on 19 November 2025 using the NCBI Datasets command-line tool [15]. A total of 2400 assemblies were initially identified.

Assemblies annotated as metagenome-assembled genomes (MAGs), highly incomplete environmental bins, or atypical *Stenotrophomonas* lineages were excluded based on assembly metadata and taxonomic screening. For the purposes of dataset construction, “atypical lineages” were defined operationally as assemblies unlikely to represent isolate genomes within the *S. maltophilia* complex. This included (i) assemblies flagged as metagenome-assembled genomes (MAGs) or environmental bins in NCBI metadata, and (ii) assemblies with taxonomic labels inconsistent with *S. maltophilia* (e.g., non-*S. maltophilia* species assignments within *Stenotrophomonas* or clear non-*Stenotrophomonas* outliers identified during taxonomic screening). These exclusions were applied prior to downstream quality control and comparative analyses. After this initial curation step, 1750 genomes were retained and downloaded (Table S2) for further processing [16,17]. Genome quality was subsequently assessed using CheckM and assembly-level metrics. Genomes were excluded if they failed any of the following criteria: completeness < 90%, contamination > 5%, more than 500 contigs, N50 < 10 kb, or ambiguous bases >1%. After quality filtering, a final dataset of 1518 high-quality genomes was retained for downstream comparative analyses.

Unless otherwise stated, all analyses including ANI clustering, PCA, core-genome phylogeny, resistome profiling, mobilome characterization, and statistical testing were performed using the final 1518-genome dataset (Table S3). A PRISMA-style flow diagram summarizing dataset construction is provided in Figure S1, and dataset usage across analyses is summarized in Table S1.

Assembly metadata from GenBank and RefSeq were merged with BioSample descriptors to extract host information, isolation source, geographic origin, and collection date. Metadata fields were further standardized and grouped into broader ecological categories, including clinical, environmental, plant-associated, animal-associated, hospital environment, and unknown. These curated metadata were used for downstream epidemiological and ecological association analyses.

Isolation source and geographic metadata were standardized using controlled vocabularies and rule-based mapping. Free-text fields from NCBI BioSample records were normalized by lowercasing, removal of punctuation, and keyword matching against curated dictionaries. Human-associated sources were mapped into standardized categories including respiratory tract, bloodstream, wound/skin, urinary tract, and other clinical sources, whereas environmental sources were grouped into water, soil, plant-associated, animal-associated, and wastewater categories. Geographic information was harmonized to country and continent levels using ISO country codes and manual correction of common spelling variants and abbreviations. Records with ambiguous, conflicting, or missing metadata were excluded from downstream association analyses.

### 2.2. Genome Quality Assessment and Filtering

Genome quality was assessed using CheckM1 v1.2.3 [16] employing the lineage-specific workflow to estimate completeness, contamination, and strain heterogeneity for each assembly. Genomes were retained only if they satisfied widely accepted criteria for high-quality draft assemblies, including ≥90% completeness and <5% contamination. Assemblies containing excessively high proportions of ambiguous bases (>1%) or more than 500 contigs were flagged and reviewed manually; genomes failing these thresholds were excluded from further analysis. The resulting high-quality dataset comprised 1518 assemblies, and the overall distribution of genome size, GC content, contig count, completeness, and contamination was summarized to characterize dataset quality.

### 2.3. Genome Annotation

All genomes were annotated uniformly using Prokka v1.14.6 [18] to ensure consistent gene prediction and functional assignment across the dataset. Genus- and species-specific annotation databases were intentionally disabled to minimize database-driven annotation bias arising from heterogeneous lineage composition, historical misannotation in public repositories, and the presence of cryptic genomospecies within the *S. maltophilia* complex. This strategy promotes consistent gene calling across divergent lineages and reduces artificial inflation or splitting of orthogroups caused by database-specific annotations, thereby improving the robustness and comparability of downstream pan-genome inference. While this approach may reduce sensitivity for assigning highly specific functional labels in some genomes, it enhances stability of gene presence–absence patterns across the full dataset. Predicted features included protein-coding sequences, tRNAs, rRNAs, and other non-coding genomic elements.

To enhance functional interpretation, predicted protein sequences were additionally annotated using EggNOG-mapper v2 [19] for COG, KEGG, and GO functional categories. Open reading frames were cross-validated using Prodigal v2.6.3 [20] when necessary.

### 2.4. Average Nucleotide Identity (ANI) Analysis and Species Delineation

Pairwise average nucleotide identity (ANI) among all representative genomes was calculated using FastANI v1.32 [14]. ANI values were used to delineate species boundaries within the *S. maltophilia* complex using a 95% ANI threshold, consistent with established species-level demarcation criteria for prokaryotes [21,22]. Genome pairs failing to meet FastANI minimum alignment fraction requirements were treated as missing comparisons and excluded from species boundary inference. This approach enabled the identification of distinct genomospecies and facilitated the detection of mislabeled genomes in the NCBI database by comparing ANI-based clusters with their originally assigned taxonomy. ANI matrices were processed to generate heatmaps and hierarchical clustering dendrograms, and ANI similarity networks were visualized using R (igraph) and Gephi v0.10.1 to characterize genomic relationships among lineages [23].

### 2.5. Pan-Genome Analysis

The pan-genome of the *S. maltophilia* complex was reconstructed using Panaroo v1.5.2 [24] in strict cleaning mode (—clean-mode strict) to minimize annotation inconsistencies and assembly artifacts. Prokka-generated GFF3 files were provided as input, and orthologous gene clustering was performed using a protein identity threshold of 95%. Paralog splitting and graph-based correction were enabled to reduce spurious gene fragmentation and misclustering arising from draft assemblies, while default strict-mode contamination filtering and cluster-merging parameters were retained.

Core genes were defined as genes present in ≥95% of genomes, with remaining genes classified as shell and cloud components, generating a comprehensive gene presence–absence matrix. Pan-genome openness was evaluated using Heaps’ law, and functional enrichment analyses of accessory genes were performed using COG and KEGG annotations. Visualization of gene frequency distributions and pan-genome accumulation curves was conducted using Panaroo utilities and custom R scripts.

### 2.6. Core-Genome Alignment and Phylogenetic Reconstruction

Core genes identified by Panaroo were aligned individually using MAFFT v7.505 [25], followed by automated trimming of poorly aligned regions using trimAl v1.5 [26]. Trimmed alignments were concatenated to generate a high-quality core-genome supermatrix. Maximum likelihood phylogenetic trees were constructed using IQ-TREE v2.2.0 [27] with ModelFinder for best-fit model selection and 1000 ultrafast bootstrap replicates to assess branch support. Phylogenetic trees were visualized and annotated in iTOL [28], incorporating metadata such as source, geography, genomospecies assignment, antimicrobial resistance (ARG) burden, and mobile genetic element profiles.

To improve readability of the phylogenetic visualization in the main manuscript, a reduced tree was generated by selecting one representative genome per GTDB classification cluster. Representative genomes were selected programmatically by retaining the first genome encountered for each unique GTDB classification label in the curated metadata table. The full maximum-likelihood phylogeny was pruned to retain only these representative tips, preserving branch lengths and topology among major lineages.

The reduced tree was used exclusively for visualization purposes in the main manuscript and does not affect any downstream analyses. The complete phylogeny including all genomes was exported as a high-resolution vector graphic and provided in the Supplementary Materials to enable detailed inspection and reproducibility.

### 2.7. Antimicrobial Resistance Gene Identification

Antimicrobial resistance genes (ARGs) were detected using AMRFinderPlus v3.11 [29] and the CARD Resistance Gene Identifier (RGI) v5.2 [30] to ensure comprehensive coverage of both intrinsic and acquired resistance determinants. Detected ARGs included multidrug efflux systems (e.g., SmeDEF and SmeVWX), β-lactamases (L1 and L2 variants), trimethoprim-sulfamethoxazole resistance genes (*sul*, *dfrA*), and other resistance factors such as aminoglycoside-modifying enzymes. ARG profiles were quantified for each genome and compared across genomospecies, phylogenetic clades, ecological sources, and geographic regions.

### 2.8. Mobile Genetic Element Profiling

The mobilome of each genome was characterized using several specialized tools. Prophages were identified using PhiSpy v4.2.21 [31] and Phigaro v2.4.0 [32], while integrative and conjugative elements (ICEs) were detected using ICEfinder v2.0. Insertion sequences (ISs) and transposases were annotated using ISEScan v1.7.3 [33], and plasmid-associated contigs were identified using mob-suite v3.1.2 [34] and PlasmidFinder v2.1.6 [35]. Mobile genetic element (MGE) abundance and diversity were compared across phylogenetic lineages and ecological categories, and correlations between MGEs and ARG burden were evaluated to identify genomic regions and lineages exhibiting elevated AMR–MGE co-occurrence within the *S. maltophilia* complex.

To account for potential assembly fragmentation bias, mobilome–ARG association analyses were adjusted for assembly quality metrics, including contig count, N50, and total genome size. Sensitivity analyses were performed using a high-contiguity subset of assemblies defined as genomes in the upper quartile of N50 and ≤250 contigs. In addition, the operational definition of “MGE-rich” genomes was evaluated across alternative percentile thresholds (top 5%, 10%, and 20% of total MGE counts). Integrated mobilome–assembly data and sensitivity datasets are provided in Tables S4–S6, and diagnostic visualization is shown in Figure S2.

### 2.9. Metadata Integration and Statistical Analyses

Associations between genomic features and metadata variables were examined using a combination of statistical approaches implemented in R v4.4.3. Fisher’s exact test or chi-square tests were applied to categorical variables, while continuous variables were compared using Wilcoxon rank-sum tests or Kruskal–Wallis tests. Multivariate analyses were performed using PERMANOVA based on Bray–Curtis distance matrices with 9999 permutations. Mantel tests were conducted using Spearman correlation with 9999 permutations. Multiple testing corrections were applied using the Benjamini–Hochberg false discovery rate method within each analysis family.

## 3. Results and Discussion

### 3.1. Genome Collection and Quality Overview

A total of 1750 *Stenotrophomonas maltophilia* genomes were retrieved from public repositories and subjected to stringent quality assessment using CheckM metrics and assembly-based thresholds. Application of minimum criteria (completeness ≥ 90%, contamination ≤ 5%, ≤500 contigs, and N50 ≥ 10 kb) resulted in a curated dataset of 1518 high-quality genomes (86.7%). The exclusion of 233 assemblies (13.3%) due to fragmentation or poor quality ensured that subsequent phylogenomic and pangenomic analyses were performed on a robust dataset. The retained genomes exhibited uniformly strong assembly characteristics, with an average completeness of 99.80% and low contamination (1.31%). These values align with recommended genome-quality thresholds for comparative genomics [16,17]. Genome size (mean 4.77 Mb) and GC content (66.38%) were consistent with established genomic features of *S. maltophilia* [1,10], further validating the reliability of the dataset for downstream analyses.

#### 3.1.1. Global Sampling Patterns Reveal Geographic and Clinical Biases

Geographic metadata revealed clear regional biases consistent with historical sequencing patterns. Most genomes originated from North America, Europe, and East Asia, with the United States contributing the largest proportion (n = 719), followed by China (n = 177), France (n = 88), Italy (n = 87), Japan (n = 63), Canada (n = 54), Spain (n = 47), and Germany (n = 46). Similar sampling imbalances have been reported in other global genomic surveys of opportunistic pathogens [1]. A total of 130 assemblies lacked geographic metadata and were annotated as Unknown, reflecting a well-recognized limitation of public repositories such as NCBI [36]. The dominance of genomes from high-income regions underscores the uneven distribution of global sequencing capacity and introduces potential bias when inferring global species diversity and lineage dynamics.

#### 3.1.2. Ecological Source Metadata Highlight Dominance of Clinical Isolates

Isolation metadata were categorized into Human, Animal, Environment, Other, and Missing. Human-associated isolates comprised the largest group (n = 976; 64.3%), predominantly derived from clinical samples such as respiratory specimens, blood, urine, and wound/skin sources. A substantial fraction was classified as “Other” (n = 335; 22.1%), reflecting non-informative or ambiguous free-text annotations that complicate ecological interpretation. Isolation source metadata were missing for 108 genomes (7.1%). This distribution reflects the prominent role of *S. maltophilia* as an opportunistic, multidrug-resistant pathogen in healthcare settings [37,38].

Animal-derived isolates were rare (n = 48; 3.2%) and included fish, livestock, and companion animals. Although comparatively uncommon, previous studies have documented the presence of *S. maltophilia* in animal hosts and suggested potential zoonotic or cross-environment interfaces [39]. Environmental isolates accounted for only 51 genomes (3.4%), despite the species being historically recognized as an environmental bacterium inhabiting soil, water, plant surfaces, and wastewater [11]. This under-representation likely reflects strong sampling and reporting bias rather than true ecological distribution. Isolation source metadata were missing for 108 genomes (7.1%), highlighting persistent inconsistencies in public metadata submission practices.

#### 3.1.3. Integration of Geographic and Ecological Patterns

**Figure 1:**
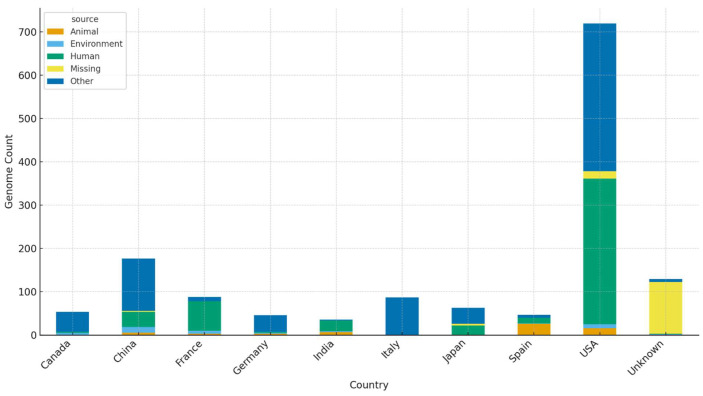
Stacked bar plot showing the distribution of 1518 high-quality *Stenotrophomonas maltophilia* genomes across the top 10 contributing countries, stratified by isolation source categories (Human, Animal, Environment, Other, Missing). Most countries are dominated by human-associated clinical isolates, whereas environmental and animal-derived genomes are comparatively rare. The “Missing” and “Other” categories highlight incomplete or non-informative metadata.

A stacked bar chart summarizing the top 10 contributing countries (Figure 1) illustrates stark differences in sampling density and ecological origins. Countries with the largest number of genomes such as the United States, China, and major European nations were heavily dominated by clinical isolates. In contrast, environmental and animal isolates were sporadic and typically represented only a small minority of genomes per country. These biases mirror trends observed in other global pathogen genomic datasets and necessitate caution when extrapolating ecological or evolutionary patterns [40].

Despite these limitations, the curated dataset provides a high-quality foundation for phylogenomic, resistome, and mobilome analyses. Recognizing the sampling and metadata biases is essential for accurate interpretation of lineage distribution, accessory genome variation, and the global evolutionary dynamics of the *S. maltophilia* complex.

### 3.2. ANI-Based Species Clusters and Misidentification

**Figure 2:**
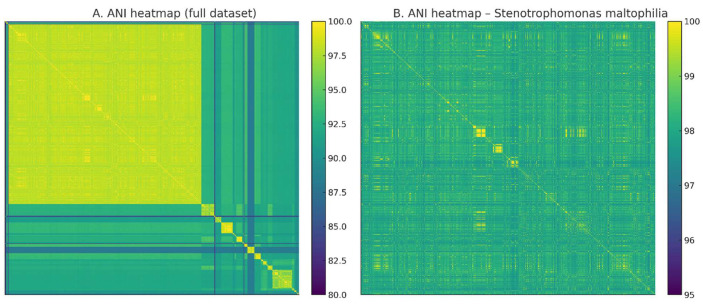
ANI heatmap and species-level genomic structure of *Stenotrophomonas*. (**A**) Full Average Nucleotide Identity (ANI) heatmap of 1518 high-quality *Stenotrophomonas* genomes, ordered according to GTDB species assignments. The block structure reveals a dominant, highly cohesive cluster corresponding to *S. maltophilia* *sensu stricto*, as well as multiple additional species-level lineages forming distinct ANI groups. (**B**) Zoomed-in ANI heatmap of the *S. maltophilia* *sensu stricto* cluster, showing uniformly high intra-lineage ANI values, with a mean pairwise ANI of 98.09% (interquartile range 97.77–98.28%), indicating strong genomic cohesion and limited within-lineage divergence. Together, these panels illustrate the extensive species-level diversity within the genus and highlight the genomic distinctiveness of *S. maltophilia* relative to other *Stenotrophomonas* species.

Average Nucleotide Identity (ANI) profiling of the 1518 high-quality *Stenotrophomonas* genomes revealed a highly structured and deeply partitioned genomic landscape (Figure 2A). ANI is widely accepted as a robust metric for bacterial species delineation, with the 95% threshold serving as the standard cut-off for species boundaries [14,22]. In line with this framework, the full ANI matrix displayed a large, cohesive cluster representing *S. maltophilia* *sensu stricto*, comprising 998 genomes that consistently exhibited intra-lineage ANI values ≥ 97%. A magnified view of this lineage (Figure 2B) further underscored its genomic uniformity, consistent with previous reports describing *S. maltophilia* as a highly conserved species despite global distribution [10,41].

**Figure 3:**
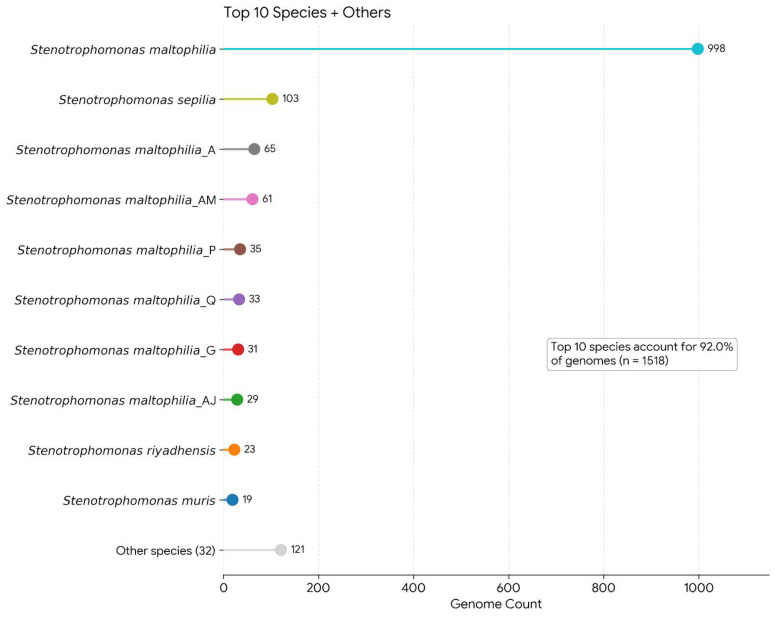
Species distribution of 1518 high-quality *Stenotrophomonas* genomes based on GTDB taxonomy. The figure shows the distribution of genomes across the top 10 GTDB-defined species clusters, with all remaining species aggregated into a single “Other species” category. *Stenotrophomonas maltophilia* *sensu stricto* represents the dominant lineage (n = 998), accounting for the majority of genomes in the dataset. The remaining genomes are distributed among multiple additional species-level clusters, including *S. sepilia*, *S. maltophilia_*A, *S. maltophilia_*AM, and other less frequent genomospecies, which together form a substantial long tail of diversity. Species are colored using a Pantone-inspired palette consistent with the ANI heatmaps and PCA analyses. This distribution illustrates the strong dominance of *S. maltophilia* *sensu stricto* while highlighting considerable species-level diversity within the genus and underscoring the prevalence of non-*S. maltophilia* genomes among publicly available assemblies.

Beyond this dominant lineage, ANI values sharply decreased below the 95% species demarcation threshold, revealing numerous divergent and well-separated groups. These clusters corresponded to GTDB-designated species such as *S. sepilia*, *S. maltophilia*_A, *S. maltophilia*_AM, *S. maltophilia*_P, *S. maltophilia*_Q, *S. maltophilia*_G, and *S. maltophilia*_AJ. The presence of these lineages reflects the expanding recognition of genomic diversity within the genus, as highlighted by recent environmental and clinical surveys. Species cluster sizes are summarized in Table 1 and visualized in Figure 3, showing that although *S. maltophilia* *sensu stricto* dominates, at least 37 additional species-level lineages are represented among public genomes.

#### 3.2.1. Extensive Misclassification of Publicly Deposited Genomes

Despite all genomes being deposited under the species name *S. maltophilia*, ANI-based delineation revealed substantial misclassification. Only 998 of the 1518 genomes (65.7%) satisfied the ≥95% ANI threshold for true *S. maltophilia* (*sensu stricto*). The remaining 520 genomes (34.3%) had ANI values of 85–94% to the *S. maltophilia* type strain and instead clustered with species such as *S. sepilia*, *S. riyadhensis*, and *S. muris*. Such pervasive taxonomic inaccuracies are consistent with previous studies highlighting widespread misannotation in GenBank and RefSeq, especially for environmentally diverse genera [16,36].

**Figure 4:**
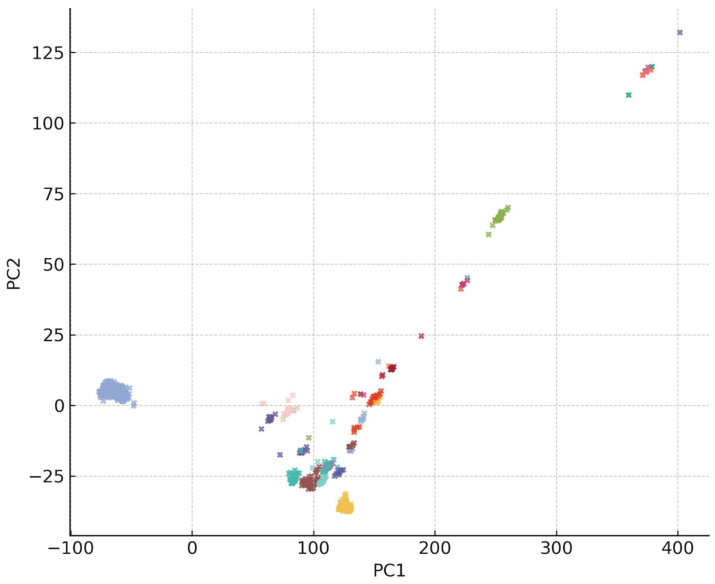
Principal component analysis (PCA) of ANI distances among 1518 high-quality *Stenotrophomonas* genomes. PCA was performed on the pairwise ANI distance matrix (distance = 100 − ANI) to visualize the genomic structure of the dataset. Each point represents a genome, colored according to its GTDB species designation using the Pantone-inspired palette applied across all ANI-based figures. *Stenotrophomonas maltophilia* *sensu stricto* forms a dense, centralized cluster, reflecting high intra-species similarity, whereas non-*S. maltophilia* genomes form distinct, well-separated groups corresponding to multiple divergent species-level clusters. The clear partitioning between species highlights the strong genomic discontinuities within the genus and supports ANI-based species delineation and correction of misidentified genomes in public databases.

Principal component analysis (PCA) of ANI-derived distances (Figure 4) corroborated the heatmap results. *S. maltophilia* *sensu stricto* formed a dense and centralized cluster, while non-*S. maltophilia* genomes separated into distinct, coherent clusters consistent with species-level divergence. Similar PCA-based stratification has been reported in other *Stenotrophomonas* genomics studies, further supporting ANI as a reliable taxonomic tool [7].

#### 3.2.2. Implications for Genomics, Clinical Diagnostics, and Surveillance

Collectively, the ANI heatmaps, species cluster structures, and PCA projections demonstrate that the *Stenotrophomonas* genus harbors far greater diversity than traditionally recognized, and this diversity has important downstream consequences. Misclassified genomes can distort comparative genomic analyses, leading to inaccurate estimates of core and accessory genome content and potentially misleading conclusions regarding metabolic traits, virulence determinants, or antimicrobial resistance features [42,43]. Taxonomic errors also affect clinical interpretation, as distinct *Stenotrophomonas* species differ in intrinsic resistance mechanisms, ecological preferences, and pathogenic potential, yet are frequently reported simply as *S. maltophilia* in diagnostic laboratories [44,45]. Furthermore, the pervasive mislabeling observed in public repositories can complicate epidemiological surveillance because incorrectly annotated genomes obscure true lineage relationships, transmission patterns, and ecological reservoirs, thereby hindering efforts to track emerging clinically enriched subclades or resistance-associated lineages [40]. These findings underscore the need for ANI-guided reannotation of public genomic databases and highlight the importance of accurate species identification for advancing genomic research, clinical diagnostics, and global pathogen surveillance.

### 3.3. Core-Genome Phylogeny and Population Structure

**Figure 5:**
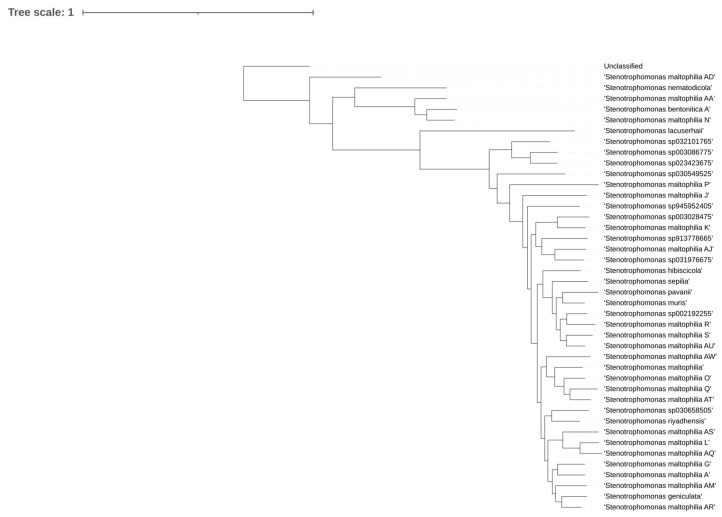
Reduced core-genome phylogeny of the *Stenotrophomonas maltophilia* complex. The tree shows one representative genome per GTDB classification cluster to improve readability and highlight major evolutionary relationships. Branch lengths are proportional to nucleotide substitutions per site. Tip labels indicate species-level assignments based on GTDB taxonomy.

The maximum-likelihood (ML) phylogeny reconstructed from the strict core-gene alignment (Figure 5 and Figure S3) revealed a highly structured population landscape within the *S. maltophilia* complex. The tree resolved 41 GTDB-designated genomospecies among the 1518 high-quality genomes, demonstrating strong agreement between core-genome evolutionary signals and the GTDB taxonomy, a relationship consistent with previous phylogenomic studies of the genus [7]. As expected, *S. maltophilia* *sensu stricto* formed the dominant and most cohesive clade, representing 998 genomes (65.7%). Additional species-level lineages such as *S. sepilia* (103 genomes), *S. riyadhensis* (23 genomes), *S. muris* (19 genomes), *S. geniculata*, and several provisional *S. maltophilia* genomospecies variants also formed monophyletic or near-monophyletic clusters, further supporting their validity as distinct evolutionary units [7]. The resolution of these strongly supported species-level clades confirms that the strict core genome provides robust phylogenetic structure and supports delineation of genomospecies within this increasingly recognized species complex.

To investigate ecological and epidemiological patterns, we overlaid isolation source, country of origin, and GTDB species assignments onto the phylogeny. Nearly half of the genomes (976, 64.3%) were annotated as human-associated clinical isolates, consistent with the species’ status as a leading multidrug-resistant opportunistic pathogen [46,47]. In contrast, only 51 genomes (3.23%) represented confidently identified environmental isolates, despite *S. maltophilia* being historically described as an environmental bacterium inhabiting soil, water, and plant-associated niches [3,48]. The skewed distribution highlights a strong sampling and reporting bias toward clinical strains, a trend noted in other global surveys of opportunistic pathogens [40]. Animal-associated isolates were rare (n = 33), but their phylogenetic positioning across the tree suggests that multiple *Stenotrophomonas* species may occasionally infect or colonize animal hosts, consistent with earlier reports of zoonotic or cross-environment occurrences [49,50].

Geographic metadata indicated broad but uneven global sampling, with large contributions from the United States, China, and multiple European countries, reflecting patterns observed in other genome-based epidemiological studies in which sequencing infrastructure is predominantly concentrated in high-income regions [36]. The interspersed distribution of countries within major clades suggests widespread global dissemination of lineages rather than strong geographic clustering, implying extensive international movement of *S. maltophilia* lineages through clinical and environmental pathways.

Visual inspection of the core-genome tree revealed several deeply branching clades within *S. maltophilia sensu stricto* that were strongly enriched for human-associated isolates, particularly those derived from respiratory and bloodstream infections. Using patristic distance-based clustering, we identified three major phylogenetically coherent sublineages embedded within the sensu stricto lineage. These sublineages represent successful, globally distributed lineages that are strongly enriched among clinical isolates, analogous to clinically important lineages described in other multidrug-resistant bacteria, such as *Pseudomonas aeruginosa* and *Klebsiella pneumoniae* [40]. The largest sublineage comprised 114 genomes and was overwhelmingly dominated by clinical isolates, with only two clearly environmental genomes. Two additional sublineages, consisting of 30 and 21 genomes, respectively, showed similar enrichment for clinical sources, reinforcing the hypothesis that these lineages are preferentially associated with hospital and human-derived settings.

Integration of MLST (Multilocus Sequence Typing) data with the phylogeny further revealed that these phylogenetic sublineages correspond to recognizable epidemiological sequence types. The largest sublineage was dominated by ST5, while the two smaller sublineages were primarily associated with ST115 and ST91, respectively, confirming earlier observations that several MLST-defined lineages exhibit global clinical prominence [51]. However, the large number of divergent genomes outside these sublineages, many of which carried rare, incomplete, or unassigned STs, illustrates the limited discriminatory power of seven-locus MLST compared to whole-genome phylogenetics, a limitation well documented in other highly diverse bacterial taxa [52]. While MLST captures broad lineage structure, the core-genome phylogeny resolves finer-scale subdivisions and provides a more accurate framework for evolutionary and epidemiological inference.

Taken together, the core-genome phylogeny, metadata-informed interpretation, and MLST integration provide a comprehensive and high-resolution view of population structure across the *S. maltophilia* complex. The delineation of genomospecies-level clades alongside a small number of globally disseminated, clinically enriched phylogenetic lineages highlights the complex evolutionary dynamics of this genus and identifies lineages of particular clinical relevance. Subsequent analyses integrate these phylogenetic patterns with resistance gene profiles, mobilome content, and ecological metadata to elucidate the genomic basis underlying the apparent success and adaptability of these emergent hospital-associated lineages.

Fine-scale phylogenetic structure in highly recombinogenic bacterial taxa can be sensitive to homologous recombination and uneven sampling density, which may influence apparent lineage boundaries and inferred ecological associations. Although our analyses reveal several lineages that are statistically enriched among clinical isolates, these patterns should be interpreted as associative rather than definitive evidence of adaptive specialization or hospital-driven selection. Future work incorporating recombination-aware phylogenetic frameworks, denser longitudinal sampling, and epidemiological linkage data will be required to robustly test hypotheses of ecological adaptation and transmission dynamics.

### 3.4. Pan-Genome Structure

Pan-genome reconstruction of the *S. maltophilia* complex revealed a highly expansive and heterogeneous genomic repertoire. Using 1518 high-quality genomes, Panaroo identified 43,355 gene families, including 1504 core genes, 1018 soft-core genes, 2241 shell genes, and a remarkably large set of 38,592 cloud genes. Core genes were defined as genes present in ≥99% of genomes. Genes present in 95–99% of genomes were classified as soft-core, those present in 15–95% as shell, and those present in <15% as cloud, following Panaroo conventions. The relatively small core genome is consistent with a collection of essential housekeeping functions that are conserved across the species complex, a pattern typical of environmentally versatile and metabolically flexible bacteria [53,54]. In contrast, the enormous cloud genome indicates extensive genomic plasticity, strain-specific gene content, and frequent horizontal gene transfer, which is a defining feature of many environmental and opportunistic pathogens [8].

The pan-genome accumulation curve increased continuously with the addition of new genomes and did not approach saturation, supporting the classification of *S. maltophilia* as having an open pan-genome. Fitting the curve to Heaps’ law (Figure S4) produced an exponent α < 1, confirming that the gene repertoire continues to expand as more genomes are sampled, a characteristic frequently observed in globally distributed environmental bacteria with large accessory gene pools [53]. The open nature of the pan-genome reflects the broad ecological range of the species, its ability to colonize diverse environmental and host-associated niches, and its continuous acquisition of novel genes from external genetic reservoirs [3,48].

Functional profiling of the accessory genome, which includes shell and cloud genes, revealed enrichment in several adaptive functional categories. COG analysis showed that accessory genes were overrepresented in amino acid transport and metabolism (COG E), carbohydrate metabolism (COG G), replication and repair (COG L), and transcriptional regulation (COG K). These categories are frequently associated with metabolic versatility, stress response, and the maintenance or mobility of genetic elements, all of which contribute to ecological adaptability and survival in fluctuating environments [54]. In contrast, core genes were dominated by essential informational and structural functions, such as translation, ribosomal assembly, and central metabolism (COG J), consistent with strong purifying selection acting on these conserved pathways.

KEGG enrichment analysis further supported the ecological adaptability of the accessory genome. Genes involved in membrane transport systems, including ABC transporters and secretion pathways, were prominent, indicating the importance of nutrient acquisition and environmental sensing. Pathways associated with xenobiotic degradation, such as aromatic compound metabolism, were enriched and reflected the species’ well-documented ability to thrive in chemically diverse or polluted environments [3]. Adaptive pathways involving nitrogen metabolism, sulfur assimilation, and two-component regulatory systems were also enriched, suggesting strong selective pressures related to nutrient limitation and environmental signaling in both natural and host-associated habitats.

Collectively, these results demonstrate that the *S. maltophilia* complex possesses a large, highly open, and functionally diverse pan-genome. The combination of a compact, conserved core genome and an extraordinarily flexible accessory genome underlines the ecological versatility of this species complex. These genomic attributes help explain its dual success as an environmental generalist and as a multidrug-resistant opportunistic pathogen capable of adapting to clinical settings, acquiring novel resistance determinants, and occupying a wide range of ecological niches.

### 3.5. Resistome Landscape Across the Species Complex

Comprehensive resistome profiling using AMRFinder revealed that antimicrobial resistance is a near-universal feature of the *S. maltophilia* complex. Among the 1518 genomes analyzed, 1517 (99.9%) encoded at least one acquired or intrinsic antimicrobial resistance gene, with a median of 10 unique ARGs per genome (interquartile range 8–16). This widespread resistance repertoire consists of the long-standing recognition of *S. maltophilia* as an intrinsically multidrug-resistant opportunistic pathogen [1,37]. Efflux-associated determinants dominated the resistome, being detected in 99.9% of genomes, followed by quinolone-associated resistance genes (98.9%) and aminoglycoside resistance genes (94.4%). Resistance to β-lactams was also highly prevalent, with 91.9% of genomes encoding at least one β-lactamase, whereas resistance genes targeting sulfonamides, tetracyclines, trimethoprim, macrolides, phenicols, and streptothricin were comparatively rare. In addition to antibiotic resistance, metal and biocide *resistance* loci were common, with nearly half of the genomes carrying copper resistance genes and approximately one-fifth harboring mercury resistance operons, reflecting the environmental origins and stress tolerance of the species complex [3,48].

Efflux systems constituted the conserved backbone of the *S. maltophilia* resistome. The tripartite MFS-type efflux module *emrABC* was nearly ubiquitous across the dataset, with *emrA*, *emrB*, and *emrC* detected in more than 98% of genomes. In parallel, the RND-type efflux determinant *smeF*, which is frequently annotated under quinolone resistance, was present in 98.9% of genomes. These efflux systems are well established as major contributors to intrinsic multidrug resistance in *S. maltophilia*, conferring reduced susceptibility to fluoroquinolones, aminoglycosides, β-lactams, and other antimicrobial classes [55,56]. Together with efflux, chromosomally encoded β-lactamases formed a second conserved pillar of resistance. The metallo-β-lactamase *bla*_L1_ was detected in 89.4% of genomes, while the serine β-lactamase *bla*_L2_ was present in 10.1%, consistent with their established roles in mediating resistance to penicillins, cephalosporins, and carbapenems in this species [57,58].

Although the intrinsic efflux–β-lactamase backbone was highly conserved, a small but clinically significant subset of genomes carried additional acquired β-lactamases. These included carbapenemases such as *bla*_NDM-1_, *bla*_VIM-1_, and *bla*_AFM-1_, extended-spectrum β-lactamases such as *bla*_GES-1_ and *bla*_GES-7_, and several OXA-type enzymes. While detected in less than 1% of genomes, the presence of these high-risk determinants is notable because *S. maltophilia* is not typically considered a major reservoir of acquired carbapenemases. Their sporadic occurrence suggests recent horizontal gene transfer events and highlights the potential role of *S. maltophilia* as a secondary reservoir or conduit for clinically important resistance genes in hospital environments [1,59].

Beyond β-lactams, aminoglycoside resistance was largely mediated by a conserved set of modifying enzymes. The phosphotransferases *aph(3′)-IIc* and *aph(6)* were detected in over 80% of genomes, while the acetyltransferase *aac(6′)-Iz* was present in approximately 42%. These enzymes are widely reported in *S. maltophilia* and contribute to reduced susceptibility to multiple aminoglycosides, often in combination with efflux mechanisms [60,61]. In contrast, classical mobile determinants conferring resistance to trimethoprim–sulfamethoxazole, the recommended first-line therapy for *S. maltophilia* infections, were rare. Sulfonamide resistance genes *sul1* and *sul2* were detected in only 3.7% of genomes, trimethoprim resistance genes *dfrA* or *dfrB* in 0.3%, and plasmid-mediated quinolone resistance gene *qnrA1* in fewer than 0.1% of genomes. These findings are consistent with previous reports indicating that resistance to trimethoprim–sulfamethoxazole remains uncommon but is emerging in localized clinical settings [4,60].

Linking resistome profiles with isolation metadata revealed a strong association between ARG enrichment and clinical origin. Genomes derived from human infections, particularly respiratory and bloodstream samples, were disproportionately represented among those with expanded ARG repertoires and carried most of the acquired high-risk determinants, including sulfonamide, trimethoprim, quinolone, and carbapenem resistance genes. In contrast, environmental and plant-associated isolates typically encoded only the intrinsic efflux systems and chromosomal β-lactamases, with little evidence of additional mobile ARGs. This pattern is consistent with hospital environments functioning as selective settings in which mobile resistance determinants are enriched in *S. maltophilia*, similar to observations reported for other opportunistic pathogens [9,62].

ARG co-occurrence network analysis further emphasized the modular organization of the resistome, a feature commonly observed in opportunistic and environmental bacteria with intrinsic multidrug resistance [63,64]. A dense and highly conserved core module was evident, centered on *emrA*, *emrB*, *emrC*, *smeF*, and *bla*_L1_, which co-occurred in the vast majority of genomes. This core was frequently linked to aminoglycoside phosphotransferases and, in nearly half of the genomes, to copper resistance genes, forming a broad multidrug and metal-resistance backbone. In contrast, peripheral network modules consisted of rarer, tightly linked acquired ARGs, including *sul1* or *sul2* paired with *dfrA* or *dfrB*, plasmid-mediated quinolone resistance genes, and acquired carbapenemases. These peripheral modules were almost exclusively confined to a small subset of clinical genomes, underscoring the sporadic and lineage-restricted patterns of clinically relevant ARG acquisition within the species complex, as reported previously for *S. maltophilia* and other hospital-associated opportunistic pathogens [65].

These results reveal a resistome architecture characterized by a highly conserved intrinsic scaffold dominated by efflux systems and chromosomal β-lactamases, overlaid with sporadic acquisition of clinically important ARGs in hospital-associated lineages. This combination of intrinsic multidrug resistance and episodic horizontal gene transfer provides a compelling genomic explanation for the persistence, adaptability, and clinical relevance of the *S. maltophilia* complex as a multidrug-resistant opportunistic pathogen.

### 3.6. Mobilome Diversity

Comprehensive mobilome profiling across the 1518 genomes of the *S. maltophilia* species complex revealed a large but highly heterogeneous repertoire of MGEs. Predictions of plasmid-associated contigs, prophages, ICEs, and ISs showed that most genomes harbored substantial mobilome content, with a median of 22 mobile elements per genome (interquartile range 16–31) and a median mobilome size of approximately 180 kb. This extensive mobilome burden is consistent with previous observations that *S. maltophilia* possesses a highly plastic genome shaped by frequent horizontal gene transfer and recombination events [3,11].

**Figure 6:**
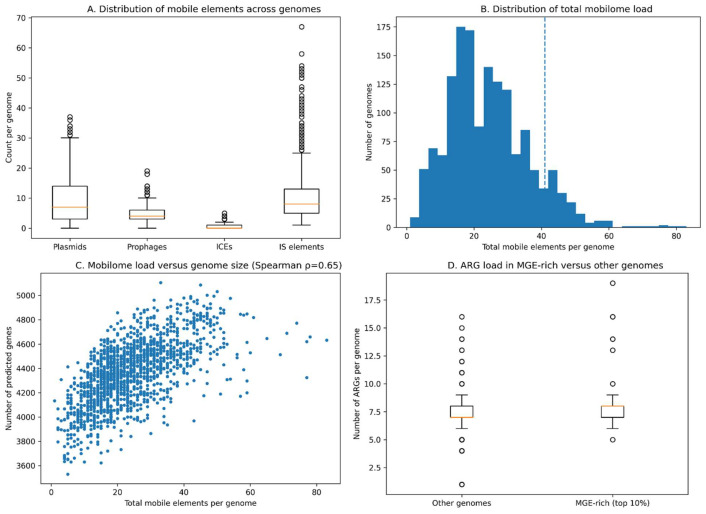
Mobilome diversity and its contribution to genome plasticity in the *S. maltophilia* complex. (**A**) Distribution of major mobile genetic element (MGE) classes per genome, including plasmid-associated contigs, prophages, integrative and conjugative elements (ICEs), and insertion sequences (ISs). IS elements representing the most abundant MGE class, followed by prophages, ICEs, and plasmid-associated regions. (**B**) Genome-wide distribution of total mobilome burden (sum of plasmids, prophages, ICEs, and ISs), highlighting a long-tailed pattern and defining MGE-rich genomes as those in the top 10% of total MGE counts (≥41 elements; dashed line). (**C**) Positive correlation between total MGE load and genome complexity, measured as the number of predicted genes per genome (Spearman’s ρ = 0.65), indicating that mobilome expansion is a major driver of accessory genome growth. (**D**) Comparison of antimicrobial resistance gene (ARG) counts between MGE-rich genomes (top 10%) and the remaining genomes, showing a higher ARG burden in mobilome-enriched lineages. The orange line in the boxplot represents the median value of the distribution.

All major MGE classes were widely distributed across the dataset, although their abundance varied markedly among genomes (Figure 6A). Prophage regions were detected in nearly all genomes, typically in multiple copies, highlighting bacteriophages as pervasive contributors to genome diversification in this species complex. ISs were numerically dominant and exhibited the greatest variability in copy numbers, reflecting their central role in mediating genome rearrangements, gene disruption, and the mobilization of adjacent genetic material [66]. ICEs were present in approximately 40% of genomes and generally occurred in low copy numbers, consistent with their episodic acquisition and stable chromosomal integration. Plasmid-associated contigs were frequently detected but often fragmented, a pattern commonly observed in short-read assemblies and in bacteria where plasmids are mosaic or partially integrated into the chromosome [67]. Together, these observations indicate that ISs and prophages constitute the core mobilome backbone of the *S. maltophilia* complex, while ICEs and plasmids contribute additional, lineage-specific variability.

The total number of MGEs per genome displayed a pronounced long-tailed distribution (Figure 6B), with approximately 10% of genomes classified as MGE-rich. When mapped onto the core-genome phylogeny, these MGE-rich genomes clustered within a limited number of well-supported lineages that were dominated by isolates of human clinical origin. In contrast, genomes belonging to predominantly environmental lineages generally harbored fewer MGEs and smaller mobilomes. This pattern suggests that hospital-associated environments are associated with elevated horizontal gene flux and the accumulation of mobile elements, as reported for other opportunistic pathogens [68].

Mobilome enrichment was closely linked to antimicrobial resistance potential. Genomes in the highest decile of total MGE counts encoded significantly larger resistomes than genomes with lower MGE burdens, carrying a median of 14 ARGs compared with 10 ARGs in the remainder of the dataset (Figure 6D). This association supports a functional coupling between MGE accumulation and the acquisition or persistence of resistance determinants, consistent with the well-established role of ICEs, plasmids, prophages, and ISs in disseminating ARGs in clinical settings [69]. These findings align with the resistome patterns described earlier (Section 3.5) and reinforce the view that mobilome dynamics are a key driver of antimicrobial resistance evolution within the *S. maltophilia* complex.

After controlling for assembly quality metrics and repeating mobilome analyses in a high-contiguity subset (n = 378 genomes), the major enrichment trends remained qualitatively consistent with the full dataset, although effect sizes were moderately attenuated, indicating partial confounding by assembly fragmentation (Figure S2; Tables S4–S7). These results confirm that the observed mobilome–ARG associations are robust and not solely driven by assembly quality artifacts.

Total MGE load was also strongly correlated with genome complexity, as measured by the number of predicted genes per genome (Spearman’s ρ = 0.65; Figure 6C). Genomes with larger mobilomes consistently encoded expanded accessory gene repertoires, indicating that prophage integration, ICE acquisition, and IS-mediated genome remodeling collectively contribute to pangenome expansion. This relationship provides a mechanistic explanation for the highly open pangenome structure observed in the *S. maltophilia* complex (Section 3.4) and supports the broader concept that MGEs are primary drivers of genomic diversification, ecological adaptation, and niche expansion in bacteria with large accessory gene pools [53].

Overall, these results demonstrate that the mobilome plays a central role in shaping genome architecture, resistance evolution, and lineage differentiation within the *S. maltophilia* species complex. The concentration of MGE-rich genomes in clinically associated lineages is consistent with hospital environments acting as selective settings for increased horizontal gene transfer and underscores the need to consider mobilome dynamics when assessing evolutionary trajectories.

#### Source- and Geography-Linked Genomic Patterns

Integration of the core-genome phylogeny with isolation source and geographic metadata revealed clear and statistically supported source- and region-associated genomic patterns across the *S. maltophilia* species complex. These patterns were tightly linked to lineage-specific mobilome expansion (Section 3.6; Figure 6) and resistome composition (Section 3.5), underscoring the combined influence of clonal population structure and horizontal gene transfer in shaping genomic diversity. Similar coupling between phylogeny, mobilome dynamics, and antimicrobial resistance has been described in other opportunistic and nosocomial pathogens [40,54].

To evaluate the robustness of source- and geography-associated patterns to metadata uncertainty, primary association analyses were repeated using a high-confidence metadata subset restricted to genomes with unambiguous isolation source assignments and validated country-level geographic annotations. The overall direction and relative magnitude of source-associated mobilome and resistome enrichment remained consistent with the full dataset, although statistical significance was moderately reduced due to the smaller sample size (Table S8). These results indicate that the observed associations are not solely driven by metadata noise or misclassification.

Clinical isolates were non-randomly distributed across the core-genome phylogeny and were disproportionately concentrated within a subset of well-supported lineages that overlapped with mobilome-rich genomes. Quantitative analysis confirmed that clinical isolates carried significantly higher mobilome burdens than non-clinical isolates (Wilcoxon rank-sum test, *p* < 0.001), consistent with the observation that clinically associated settings are associated with elevated horizontal gene flux, although causality cannot be inferred from these observational data. In contrast, genomes derived from environmental sources were more evenly distributed across the phylogeny and typically harbored fewer mobile genetic elements, suggesting lower rates of recent gene acquisition outside host-associated settings. These findings align with previous reports indicating that antimicrobial exposure and dense microbial communities in clinical environments are consistent with strong selective pressures favoring mobile element accumulation [69].

Clear functional differences further distinguished clinical and environmental genomes. Clinical isolates encoded significantly higher numbers of both mobile genetic elements and antimicrobial resistance genes, reflecting coordinated mobilome enrichment and resistome expansion. In particular, genomes carrying integrative and conjugative elements were predominantly of clinical origin and harbored significantly larger ARG repertoires than ICE-negative genomes (Wilcoxon rank-sum test, *p* < 0.001; Figure 6D). Environmental isolates, by contrast, generally encoded only the conserved intrinsic resistance backbone and maintained smaller accessory genomes, indicative of a more stable genomic architecture shaped primarily by vertical inheritance rather than frequent horizontal exchange. This pattern supports earlier observations that ICEs and related MGEs play a central role in disseminating resistance determinants in hospital-associated bacteria [69,70].

Geographic origin also contributed to structuring genomic diversity within the species complex. Mobilome burden differed significantly across continents (Kruskal–Wallis test, *p* < 0.01), with isolates from Asia exhibiting the highest total MGE counts, followed by those from Europe and the Americas. Despite these quantitative differences, dominant insertion sequence families and conserved prophage types were shared across regions, indicating global dissemination of successful mobile elements. This pattern suggests that region-specific mobilome expansion is superimposed on a globally circulating pool of MGEs, a phenomenon commonly observed in widespread opportunistic pathogens with open pangenomes [40,54].

Multiple lines of evidence collectively support the presence of clinically enriched genomic subclades within the *S. maltophilia* complex. These clusters are characterized by tight phylogenetic grouping, enrichment for clinical isolates, elevated mobilome burdens, and increased ARG content. Notably, several such clusters comprised isolates originating from multiple countries and continents while sharing highly similar mobilome profiles, consistent with recent clonal expansion and interregional dissemination. Together, these findings indicate that hospital environments are consistently associated with mobilome-enriched, multidrug-resistant *S. maltophilia* lineages, reinforcing the value of genome-based surveillance frameworks that integrate phylogeny, mobilome dynamics, and resistance profiling.

Public genome repositories exhibit substantial geographic and source sampling biases, with over-representation of clinical isolates from a limited number of countries. Although we performed sensitivity analyses using high-confidence metadata subsets, residual sampling bias may still influence apparent continental or source-associated patterns. Consequently, geographic and ecological associations should be interpreted cautiously and primarily as descriptive trends rather than definitive evidence of population structuring.

Several limitations should be considered when interpreting these findings. First, the isolation source and geographic metadata derived from public repositories remain incomplete and heterogeneous despite extensive standardization efforts, which may introduce classification uncertainty and reduce statistical power in subset analyses. Second, the dataset exhibits pronounced geographic and sampling bias toward regions with intensive clinical sequencing activity, potentially limiting generalizability of global population structure and ecological inferences. Third, the majority of genomes analyzed were short-read draft assemblies, which can fragment plasmids, integrative conjugative elements, and prophages, thereby limiting accurate reconstruction of mobile genetic element architecture and copy number. Although sensitivity analyses were performed to mitigate assembly-related bias, long-read or hybrid assemblies will be essential for more precise characterization of mobilome dynamics in future studies.

## 4. Conclusions

This study represents the largest and most comprehensive comparative genomic analysis of the *Stenotrophomonas maltophilia* species complex conducted to date, integrating 1518 high-quality genomes from diverse ecological, geographic, and clinical origins. By combining ANI-based species delineation, core-genome phylogenomics, pan-genome reconstruction, and detailed resistome-mobilome profiling, we provide a robust and unified framework for understanding the evolutionary structure and adaptive dynamics of this clinically important yet taxonomically complex pathogen.

Our analyses clarify long-standing taxonomic ambiguities by demonstrating that more than one-third of publicly available genomes labeled as *S. maltophilia* are misclassified and instead belong to distinct genomospecies within the broader complex. These findings highlight the urgent need for ANI-guided reannotation of public databases and underscore the limitations of conventional identification methods in both research and clinical diagnostics. Accurate species-level classification is essential, as distinct lineages exhibit markedly different resistance profiles, mobilome content, and ecological associations.

The *S. maltophilia* complex was shown to possess a highly open pan-genome, characterized by a compact conserved core and an exceptionally large, functionally diverse accessory genome. This genomic architecture reflects extensive horizontal gene transfer and underpins the species complex’s remarkable ecological versatility. Mobilome analyses revealed that insertion sequences and prophages form a pervasive backbone of genome plasticity, while integrative and conjugative elements and plasmid-associated regions contribute lineage-specific variation and is consistent with key vehicles for antimicrobial resistance dissemination.

Resistome profiling demonstrated that intrinsic multidrug resistance dominated by efflux systems and chromosomal β-lactamases is nearly universal across the complex, while the acquisition of clinically important resistance determinants, including sulfonamide, trimethoprim, and carbapenem resistance genes, remains relatively rare but is strongly concentrated in hospital-associated lineages. The tight coupling between mobilome enrichment and expanded resistomes in these lineages provides compelling genomic evidence consistent with clinical environments acting as selective settings for elevated horizontal gene transfer and resistance evolution.

Integration of phylogeny with ecological and geographic metadata further revealed non-random source- and region-associated genomic patterns, including the presence of globally disseminated, clinically enriched phylogenetic lineages characterized by elevated mobilome burdens and resistance gene content. These findings support the presence of globally disseminated, clinically enriched lineages within *S. maltophilia sensu stricto* and emphasize the importance of genome-resolved surveillance approaches for tracking their emergence and spread.

Collectively, this study establishes a curated, reference-quality genomic framework for the *S. maltophilia* species complex and provides critical insights into its taxonomy, population structure, and resistance evolution. The resources and evolutionary context generated here lay a strong foundation for future national and international genomic surveillance, risk assessment, and infection-control strategies and will facilitate more accurate interpretation of clinical, environmental, and epidemiological data for this increasingly significant multidrug-resistant opportunistic pathogen.

## Figures and Tables

**Table 1:** GTDB species clusters among 1518 high-quality genomes.

GTDB Species	Genome Count	Interpretation
*Stenotrophomonas maltophilia*	998	True *S. maltophilia* *sensu stricto* lineage; high intra-species ANI (≥97%)
*Stenotrophomonas sepilia*	103	Distinct species cluster; misidentified as *S. maltophilia* in NCBI
*S. maltophilia*_A	65	Divergent species-level cluster below 95% ANI
*S. maltophilia*_AM	61	Divergent species; often incorrectly labeled *S. maltophilia*
*S. maltophilia*_P	35	Subspecies-level clade; <95% ANI to type strain
*S. maltophilia*_Q	33	Divergent clade; ANI supports species-level status
*S. maltophilia*_G	31	Distinct species-level lineage
*S. maltophilia*_AJ	29	Divergent lineage; misannotated genomes
*Stenotrophomonas riyadhensis*	23	Recognized species separate from *S. maltophilia*
*Stenotrophomonas muris*	19	Distinct species; not *S. maltophilia*
Additional species cluster (n = 28)	121 combined	Smaller species groups represented by ≤15 genomes each
Total	1518	High-quality genomes retained after QC filtering

## Data Availability

A consolidated genome-level metadata table including accession identifiers, assembly metrics, original NCBI taxonomy, GTDB classification, ANI cluster membership, curated source and geographic annotations, and summary resistome and mobilome features is provided as Supplementary Table S9. To ensure full reproducibility, the complete computational workflow, including genome retrieval, quality control, annotation, pan-genome analysis, phylogenetic reconstruction, resistome detection, mobilome profiling, and statistical analyses, is provided as Supplementary Scripts S1–S9, together with configuration files and documented command-line parameters.

## Supplementary Materials

The following supporting information can be downloaded at: https://www.mdpi.com/article/10.3390/life16010158/s1, Figure S1: PRISMA-style workflow for genome dataset construction and analysis; Figure S2: MGE count vs Assembly fragmentation; Figure S3: The maximum-likelihood (ML) phylogeny of *S. maltophilia* complex; Figure S4: Pan-genome accumulation and Heaps’ law fit; Table S1: Dataset usage; Table S2: Final dataset metadata; Table S3: Final QC data; Table S4: MGE merge metrics; Table S5: MGE high contiguity subset; Table S6: MGE thresholds; Table S7: MGE correlations; Table S8: Metadata sensitivity with ARGs; Table S9: Master genome metadata with curated country; Supplementary scripts.
