# Supplementary figures and images for: Global Comparative Genomics of Stenotrophomonas maltophilia Reveals Cryptic Species Diversity, Resistome Variation, and Population Structure
Source: Life (Basel). 2026 Jan 17;16(1):158. doi: 10.3390/life16010158 (PMC12842811; doi:10.3390/life16010158)

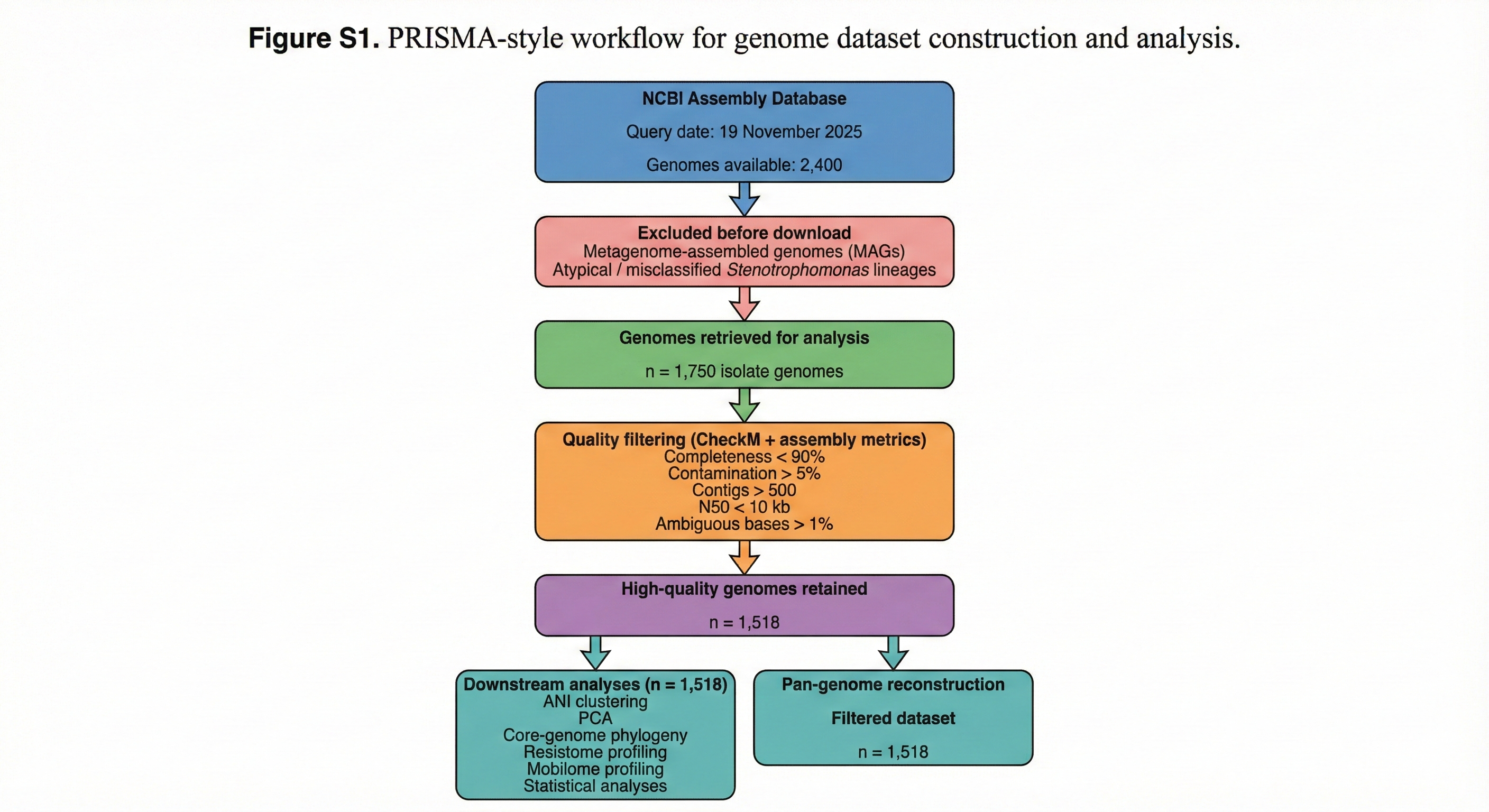

Supplement: Supplementary file 1 [file life-16-00158-s001.zip › FigureS1.png]

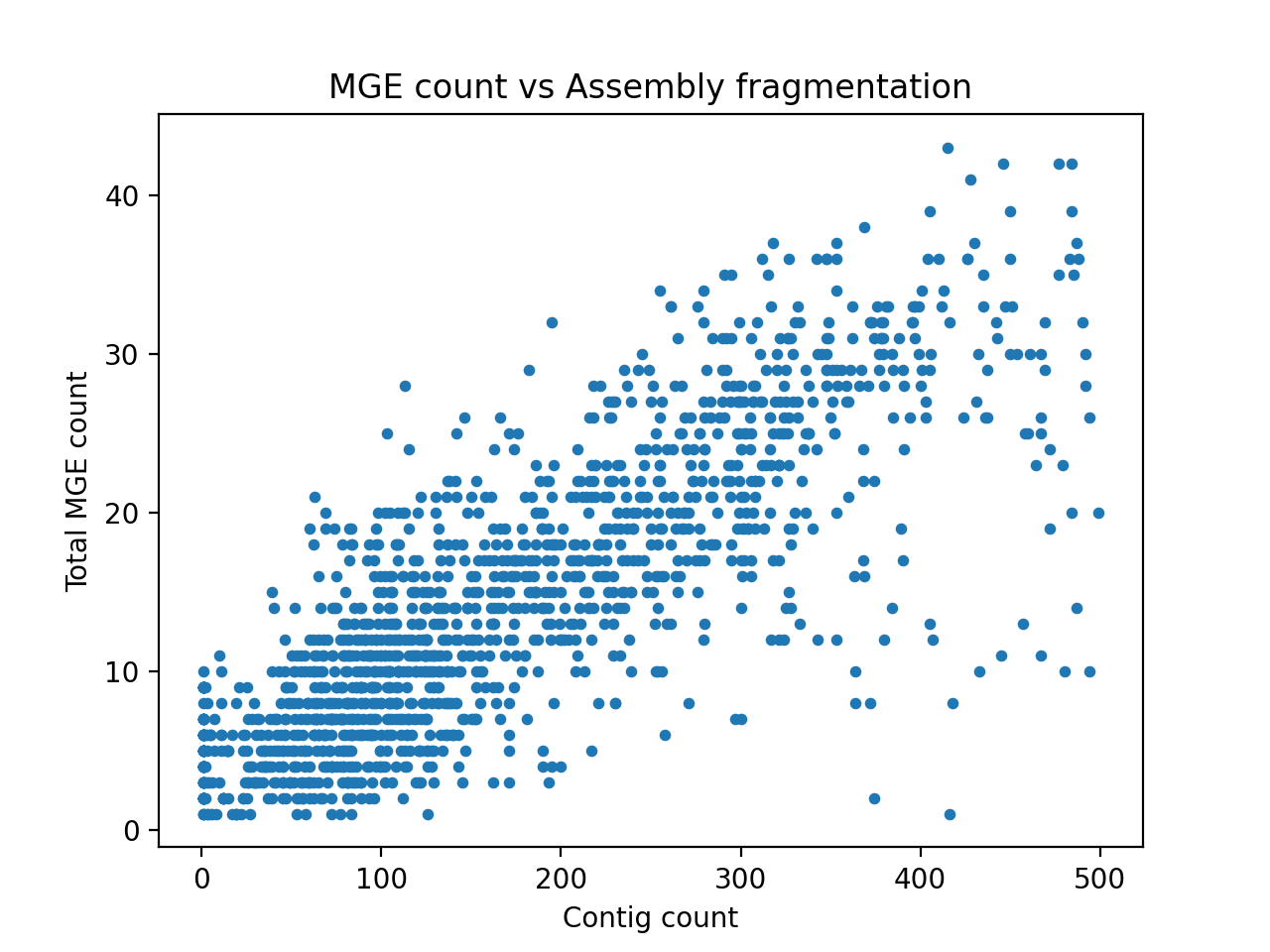

Supplement: Supplementary file 1 [file life-16-00158-s001.zip › FigureS2.png]

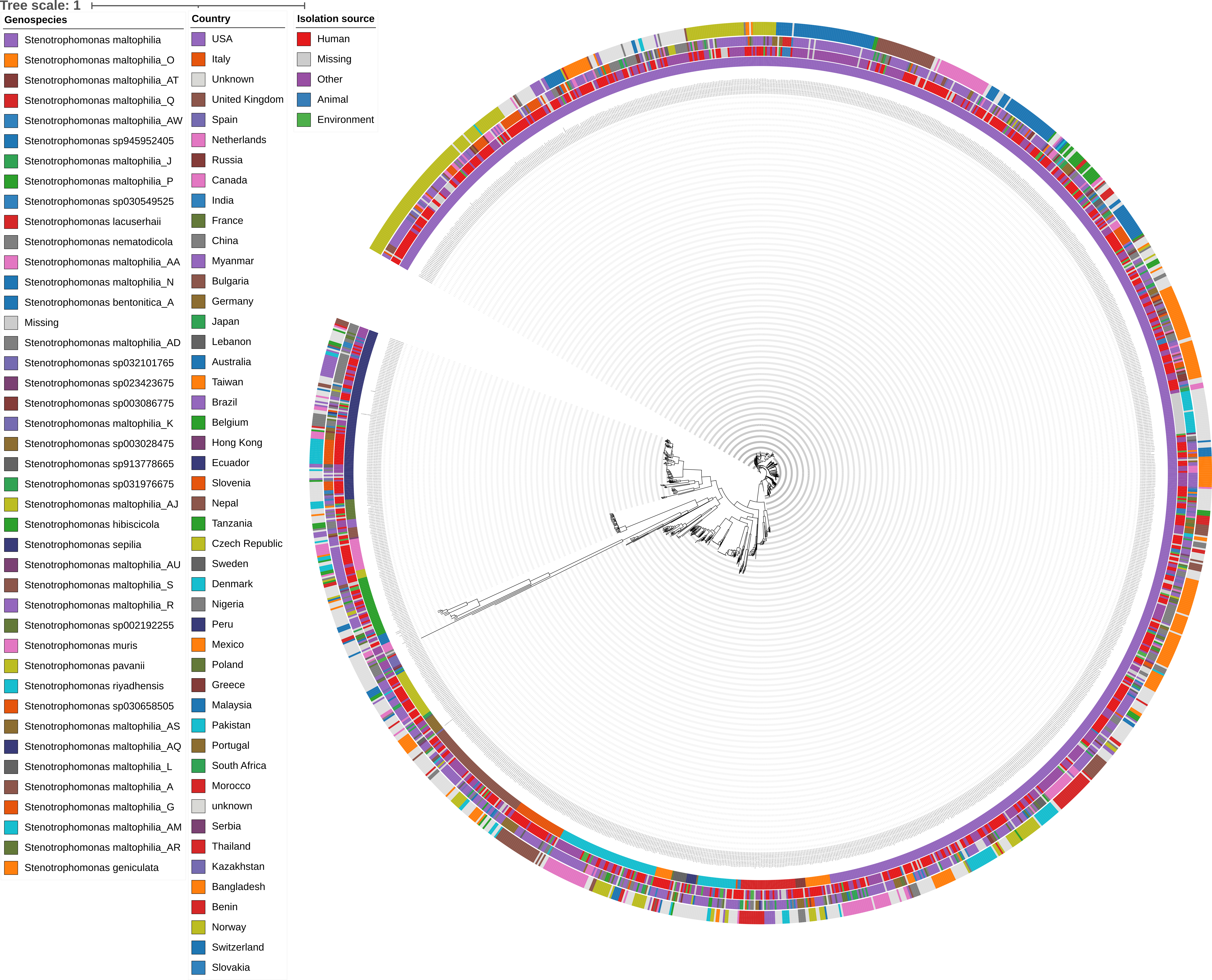

Supplement: Supplementary file 1 [file life-16-00158-s001.zip › FigureS3.png]

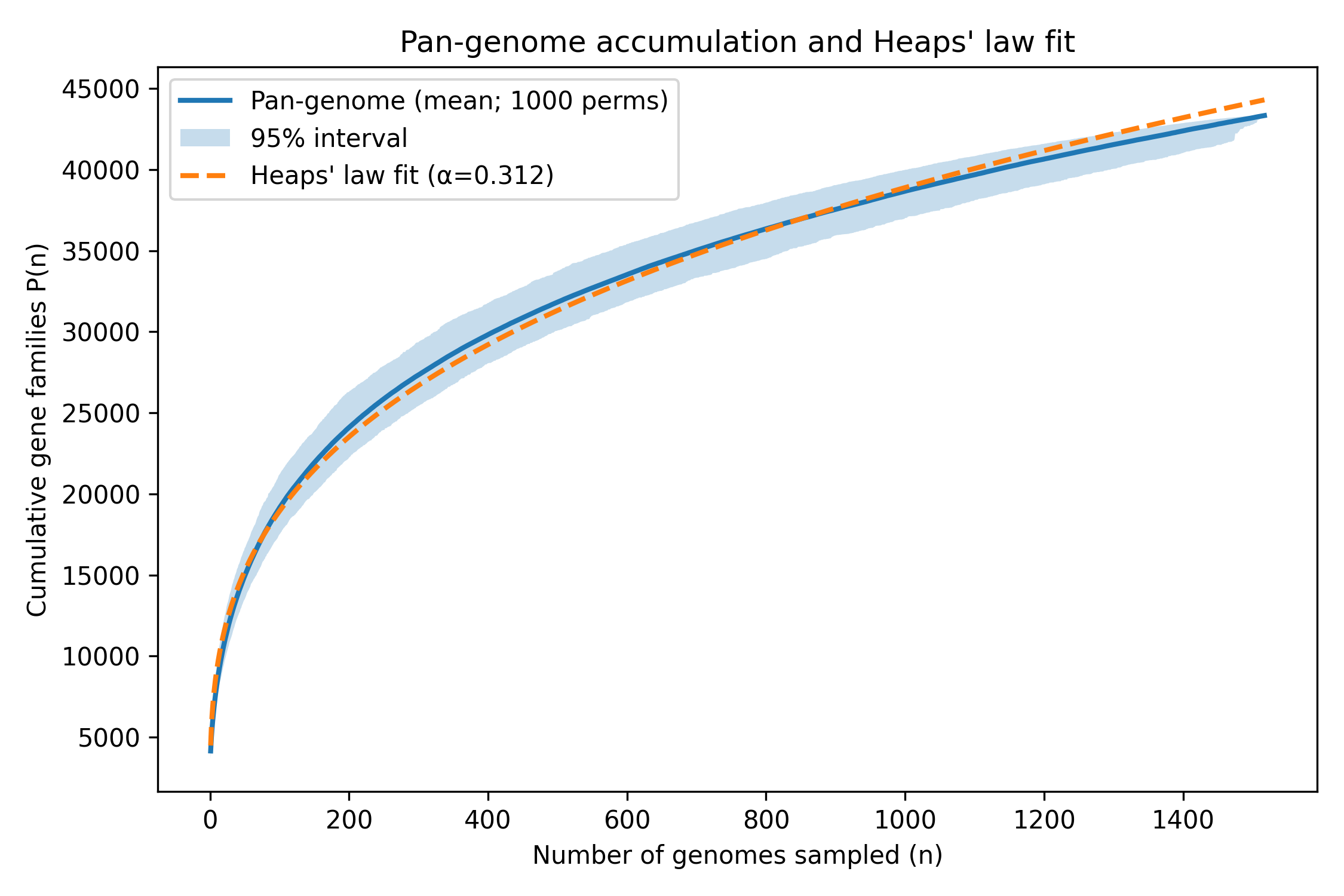

Supplement: Supplementary file 1 [file life-16-00158-s001.zip › FigureS4.png]
